# Is vitamin D deficiency involved in the immune reconstitution inflammatory syndrome?

**DOI:** 10.1186/1742-6405-6-4

**Published:** 2009-04-21

**Authors:** Anali Conesa-Botella, Chantal Mathieu, Robert Colebunders, Rodrigo Moreno-Reyes, Evelyne van Etten, Lut Lynen, Luc Kestens

**Affiliations:** 1 Institute of Tropical Medicine, Department of Clinical Sciences, Antwerp, Belgium; 2 Katholieke Universiteit Leuven, Laboratory of Experimental Medicine and, Endocrinology, Leuven, Belgium; 3 University of Antwerp, Faculty of Medicine, Antwerp, Belgium; 4 Université Libre de Bruxelles, Department of Nuclear Medicine, Brussels, Belgium; 5 Institute of Tropical Medicine, Department of Immunology, Antwerp, Belgium

## Abstract

**Background:**

About 20–30% of persons with HIV infection, especially those living in countries with limited resources, experience an immune reconstitution inflammatory syndrome (IRIS) after starting antiretroviral treatment. The active form of vitamin D, 1,25-dihydroxyvitamin D, is a key player in the clearance of pathogens and influences the level of inflammation and macrophage activation.

**Presentation of the hypothesis:**

We hypothesize that low availability of 1,25-dihydroxyvitamin D, either due to vitamin D deficiency or due to polymorphisms in the vitamin D receptor or in its activating/inactivating enzymes, contributes to the appearance of IRIS. Furthermore, drug interactions with the enzymatic pathways of vitamin D could favour the development of IRIS.

**Testing the hypothesis:**

Our hypothesis could be explored by a case-control study to assess the prevalence of vitamin D deficiency in HIV-infected patients on antiretroviral treatment who develop and do not develop IRIS.

**Implications of the hypothesis:**

If the role of vitamin D in IRIS is confirmed, we would be able to screen patients at risk for IRIS by screening for vitamin D deficiency. After confirmation by means of a clinical trial, vitamin D supplementation could be a cheap and safe way to reduce the incidence of IRIS.

## Background

Highly active anti-retroviral therapy (HAART) decreases the mortality and improves the quality of life of persons living with human immunodeficiency virus (HIV) infection [1]. Nevertheless, 17–32% of HIV infected persons living in countries with limited resources experience a temporary worsening of their clinical status after starting HAART despite immunological improvement [2,3]. This paradoxical reaction occurs most frequently during the first 3 months after initiation of HAART and is known as immune reconstitution inflammatory syndrome (IRIS) or immune restoration disease (IRD) [4]. To date, more than 20 different pathogens have been associated with IRIS [2,3,5,6]. However, IRIS has also been described in association with autoimmune diseases, cancer, and some non-infectious granulomatous diseases such as sarcoidosis and Crohn's disease [7].

In countries with limited resources, Mycobacteria sp. are by far the most common pathogens involved [5].

There is now evidence that vitamin D plays a role in improving anti-tuberculosis immunity as well as in the regulation of immune responses [8-11], both of which are crucial steps in the development of IRIS. A double blind randomized controlled trial showed that a single dose of vitamin D significantly enhanced immunity to *Mycobacteria tuberculosis *(Mtb) among contacts of tuberculosis (TB)-infected patients [12]. Liu et al showed later that vitamin D acts by increasing the level of the antimicrobial peptide cathelicidin produced by monocytes and macrophages [13,14].

Low levels of vitamin D levels have been observed in African populations [15] as well as in HIV-infected persons (reviewed by Villamor [16]). A recent study in a cohort of HIV-positive patients in the Netherlands (73% white, 20% black) showed a prevalence of vitamin D deficiency of 29% in the total population, and 62% in black patients. Low levels of active vitamin D have been associated with low CD4 counts and AIDS progression [17].

TB treatment is also known to interfere with vitamin D metabolism and to cause osteomalacia [18]. Vitamin D deficiency may be influenced by deficient substrate, but also by polymorphisms in its receptor or in the enzymes controlling the activation of this steroid.

## Presentation of the hypothesis

Low levels of vitamin D could predispose HIV infected patients with a current or undiagnosed opportunistic infection (OI) to IRIS. Indeed, the active form of vitamin D, 1,25-(OH)_2_D, has anti-inflammatory activity [19] and there is now accumulating evidence for its role in the regulation of human T-cell and antigen-presenting cell (APC) functions [20,21]. Furthermore, drug interactions with the enzymatic pathways of vitamin D [22] could favour the development of IRIS.

### Pathogenesis of IRIS

HIV causes progressive depletion of CD4+ T-cells and impairs the immune system [2,5]. In HIV/Mtb patients with severe immunodeficiency, impaired T-cell function impedes granuloma formation [23]. When HAART is started, T-cell function is restored and granuloma formation is re-established, mainly in the lungs and lymph nodes, through activation of Mtb-infected macrophages by interferon-γ (IFN-γ) producing T-cells [23]. Unfortunately, rapid or unbalanced restoration of the immune system against living or death organisms [7,24] may also lead to uncontrolled antigen-specific responses [2] with reappearance of clinical symptoms [5] and development of IRIS.

Known risk factors for the development of IRIS include a low CD4 T-cell count when starting HAART, advanced OI with high OI antigen load, and a short time interval between OI treatment and the start of HAART [2,25-28]. Other risk factors such as younger age, male gender, a higher CD8 T-cell percentage, a high viral load at baseline, a fast increase in CD4 T-cell count and fast decrease in viral load after the start of HAART, and a protease inhibitor (PI) based HAART regimen, were reported in some studies but not in others [29].

### Vitamin D and the immune system

The main source of vitamin D stems from sun exposure: pre-vitamin D is converted by solar ultraviolet B radiation in the skin into vitamin D. Skin pigmentation is a known risk factor for hypovitaminosis D since melanin, responsible for the skin pigmentation, filters UV radiation [30-32].

**Figure 1:**
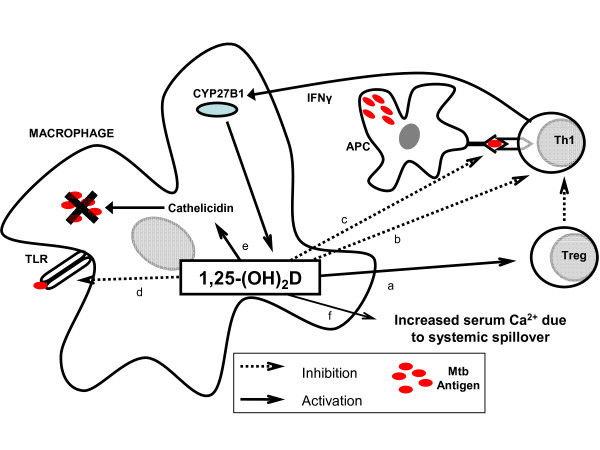
**Role of vitamin D locally at the inflammation site – example based on Mtb infection**. 1,25-(OH)_2_D, the active form of vitamin D produced by macrophage-CYP27B1 at the inflammation site, has many local actions leading to a negative feedback loop avoiding macrophage overstimulation. 1,25-(OH)_2_D reduces T helper (Th1) lymphocyte-mediated macrophage activation, (a) by activating regulatory T-cells (Treg) which inhibit the activation of Th1 lymphocytes by antigen-presenting cells (APC) [36], (b) by directly inhibiting activation of Th1 lymphocytes and thus their interferon-γ (IFN-γ) production, and (c) by preventing antigen presentation by APC to Th1 lymphocytes [34]. 1,25-(OH)_2_D acts also directly on macrophages (d) by reducing expression of Toll-like receptor (TLR) to *Mycobacterium tuberculosis *(Mtb) [34], and (e) by inducing intracellular Mtb destruction via the cathelicidin-mediated system. If macrophages are overstimulated, high local level of 1,25-(OH)_2_D could lead to systemic spill over and thus hypercalcemia, as has been described in Mtb-IRIS [39], since no systemic negative feedback by the parathyroid axis exists on macrophage-1,25-hydroxylase (CYP27B1) [15].

Food uptake is limited to vitamin D supplementation or consumption of oily fish [31]. Vitamin D is transported into the blood by the vitamin D-binding protein (VDBP) is converted in the liver by 25-hydroxylases (CYP34A, CYP27A1, CYP2R1, ...) into 25-hydroxyvitamin D (25-(OH)D). 25-(OH)D is considered the best indicator of vitamin D status [31,33] with normal levels between 30 and 50 ng/ml [34]. Vitamin D deficiency is defined as 25-(OH)D below 20 ng/ml [31]. The cytochrome CYP3A4, present in liver, intestine, kidney and leukocytes [35] is also a key enzyme in P450 cytochrome-mediated drug metabolism such as anti-retrovirals (non nucleoside reverse transcriptase inhibitor and PI) as well as certain anti-tuberculous drugs (rifampicin and isoniazid) [18,33,35]. The inactive form of vitamin D, 25-(OH)D, is converted in kidney cells into its circulating active form 1,25-(OH)_2_D by the enzyme 1-α-hydroxylase (CYP27B1). Other cells such as macrophages also express CYP27B1 [33]. In the late phase of macrophage activation, macrophage-CYP27B1 produces 1,25-(OH)_2_D which presumably has a local rather than a systemic effect on immune cells [20]. Although the macrophage-CYP27B1 is identical to the renal CYP27B1, its expression is not down-regulated by the parathyroid hormone nor the active vitamin D and is mainly up-regulated by inflammatory cytokines such as IFN-γ and by lipopolysaccharides (LPS) [20,33]. Figure 1 illustrates the complex action of active vitamin D on regulatory and effector T-cells and on APC, which results in a negative feedback on macrophage activation to prevent their overstimulation [20,36].

To exert its actions, 1,25-(OH)_2_D binds to the vitamin D receptor (VDR) expressed in normal, malignant and immune cells [34]. At least 36 tissues possess VDR and more than 10 tissues are able to produce 1,25-(OH)_2_D in a paracrine fashion [8]. By the wide expression of VDR [8], 1,25-(OH)_2_D can regulate calcium homeostasis and bone metabolism as well as play an essential role in cell proliferation, differentiation and the above described regulation of the immune response [31,33]. The variability in patients' susceptibility to immune dysfunction and thus IRIS could be explained by the polymorphisms of the VDR gene known to influence immune cell function [37], or by polymorphisms of hydroxylases regulating the production or the degradation of the bioactive vitamin D.

### Vitamin D, HAART and IRIS

During HAART and immune reconstitution, pathogen-derived antigens are de novo recognized by APC, processed and presented to CD4+ T-cells leading to T-cell activation and secretion of macrophage-activating interferon-γ. Vitamin D inhibits this IFN-γ production [34].

In case of low 25-(OH)D levels prior to HAART, we hypothesize that a defective clearing of pathogens and a delayed negative feedback on macrophage activation due to low 1,25-(OH)_2_D production, can lead to excessive granuloma formation and an exacerbated inflammatory response described as IRIS.

To avoid macrophage-overstimulation, vitamin D should be given before their massive activation, i.e. before the initiation of the inflammation. Indeed vitamin D decreases immune stimulation [38], but if vitamin D is given when granulomas are already flourishing, the 1α-hydroxylase in activated macrophages can produce high amounts of 1,25-(OH)_2_D with systemic spillover [34] resulting in hypercalcemia [38] as described in Mtb-IRIS [39-41] and cryptococcus-IRIS [42]. At that moment vitamin D supplementation could worsen the clinical status of the patient.

**Figure 2:**
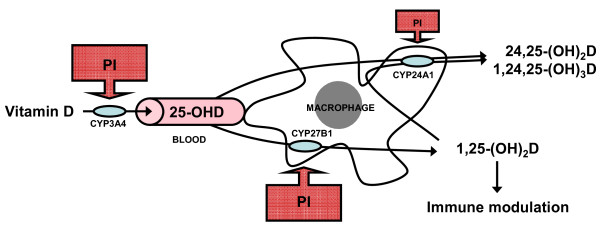
**Vitamin D production in macrophages and PI interaction**. The 25-hydrolylase CYP3A4 converts vitamin D into 25-(OH)D, its inactive form. To be active, the circulating 25-(OH)D is 1-α-hydroxylated by the renal or the extrarenal P450 cytochrome (CYP27B1) into 1,25-(OH)_2_D. Both 25-(OH)D and 1,25-(OH)_2_D can also be catabolized by 24-hydroxylation (CYP24A1) into 24,25-(OH)_2_D and 1α,24,25-(OH)_2_D respectively [33]. Activated macrophages possess both CYP27B1 and CYP24A1 and are able to produce 1,25-(OH)_2_D locally at the site of inflammation. Protease inhibitors (PI) inhibit the function of the hepatic-CYP3A4 and the macrophage-CYP27B1 which are critical for active vitamin D synthesis, and exert a milder inhibition on the activity of the 24-hydroxylase (arrows). The net effect is a reduced production of 1,25-(OH)_2_D [22] that could influence immunity.

Protease inhibitors (PI) are known to interfere with vitamin D metabolism (Figure 2) reducing 1,25-(OH)_2_D levels [22]. Low 1,25-(OH)_2_D levels and bone loss has been described to be most frequent in PI-treated patients compared to other HAART regimens [43,44]. Brown et al. concluded that odds of having osteoporosis was 1.6 times higher if patients where PI-treated [45]. We suggest that the interaction between PI and vitamin D metabolism could result in an increased risk of IRIS in patients treated with a PI-based regimen [29].

## Testing the hypothesis

Our hypothesis could be explored by a case-control study to assess the prevalence of vitamin D deficiency in HIV infected patients on HAART who develop and do not develop IRIS. In cohort studies of patients initiated on HAART, the incidence of IRIS should be compared in patients with low, normal and high baseline vitamin D levels.

Moreover, polymorphisms of the VDR, the VDBP and the enzymes involved in vitamin D production should be investigated. We also propose to perform functional testing of vitamin D enzymes to find out if an increase of the vitamin D catabolism or a decrease of its production could contribute to low 1,25-(OH)_2_D concentrations at the site of inflammation, possibly leading to IRIS. Finally remains the issue whether the levels of vitamin D that are currently accepted as 'sufficient' for bone health apply to 'global health' and in particular anti-bacterial and anti-inflammatory properties of vitamin D [46].

## Implication of the hypothesis

There is an inter-relationship between vitamin D metabolism, HAART therapy and immunity. Impaired vitamin D metabolism in macrophages, whether caused by vitamin D deficiency or by HAART therapy, might be a determinant of IRIS in HIV-positive individuals. The potential role of vitamin D status in the pathogenesis of IRIS should be investigated. Indeed, if the role of vitamin D in IRIS is confirmed, vitamin D supplementation could be a cheap and safe way to prevent IRIS.

## Competing interests

The authors declare that they have no competing interests.

## Authors' contributions

ACB wrote the paper. CM, RC, RMR, EvE, LL and LK participated in developing the hypothesis and collaborated in the writing and reviewing of the article.
